# Multivessel Woven Coronary Artery Disease

**DOI:** 10.21470/1678-9741-2021-0153

**Published:** 2021

**Authors:** Luis Roberto Palma Dallan, Luís Alberto Oliveira Dallan, Miguel Moretti, Ana Beatriz Camerlengo Moragas, Luís Augusto Palma Dallan, Fabio B.Jatene

**Affiliations:** 1 Department of Cardiovascular Surgery, Instituto do Coração, Hospital das Clínicas, Faculdade de Medicina, Universidade de São Paulo, São Paulo, São Paulo, Brazil.; 2 Department of Cardiology, Instituto do Coração, Hospital das Clínicas, Faculdade de Medicina, Universidade de São Paulo, São Paulo, São Paulo, Brazil.; 3 Faculdade de Medicina de Marília, Marília, São Paulo, Brazil.; 4 Department of Interventional Cardiology, Instituto do Coração, Hospital das Clínicas, Faculdade de Medicina, Universidade de São Paulo, São Paulo, São Paulo, Brazil.; 5 Department of Cardiovascular Medicine, Harrington Heart and Vascular Institute, University Hospitals Cleveland Medical Center, Cleveland, Ohio, United States of America.

**Keywords:** Coronary Artery Disease, Woven Coronary Disease, Coronary Anomaly, Coronary Artery Bypass Grafts

## Abstract

Woven coronary disease is a rare pathology with unknown etiology. Although initially considered benign, recent publications report myocardial ischemia caused by the affected vessel. Since most patients are asymptomatic, long-term follow-up to understand its behavior is mandatory. We report a multivessel woven disease case with documented ischemia that was submitted to coronary artery bypass grafting and remained asymptomatic for two years of follow-up.

**Table:** 

Abbreviations, acronyms & symbols	
CABG	= Coronary artery bypass grafting
CX	= Circumflex artery
Dg	= Diagonal branch of the LAD
LAD	= Left anterior descending artery
LIMA	= Left internal mammary artery
PCI	= Percutaneous coronary intervention
RCA	= Right coronary artery

## INTRODUCTION

A 40-year-old male patient had symptoms of dyspnea on moderate exertion and chest discomfort. The patient was a nonsmoker and had no other risk factors for coronary disease. He had been previously hospitalized for minor stroke without cognitive or motor impairment. His complementary diagnostic exams at the stroke event were magnetic resonance imaging demonstrating acute ischemia on his right cerebellar artery; angiotomography showing right vertebral artery tapered with areas of diffuse stenosis; and doppler ultrasound demonstrating right carotid plaque.

**Fig. 1:**
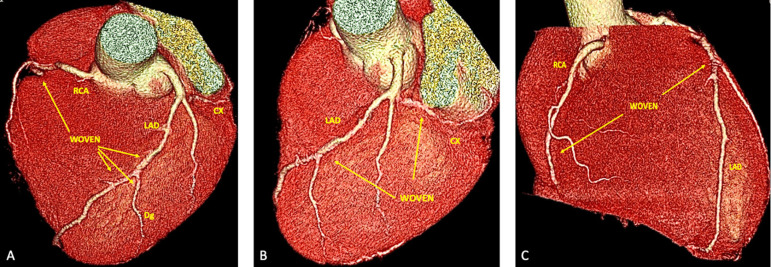
Angiotomography - Multivessel woven coronary artery disease. A) Woven coronary disease affecting LAD on its proximal and middle thirds, proximal Dg. B) LAD affected on its proximal third and total occlusion of the CX. C) RCA affected on its middle and distal thirds and LAD af fected on its middle third. CX=circumflex artery; Dg=diagonal branch of the LAD; LAD=left anterior descending artery; RCA=right coronary artery.

Initial cardiologic investigation presented exercise stress test positive for ischemia. His echocardiogram showed ejection fraction of 63% with normal ventricular wall motion, without valvular commitment. Angiotomography showed intraluminal filling defect on all three major coronaries (Figure 1).

**Fig. 2:**
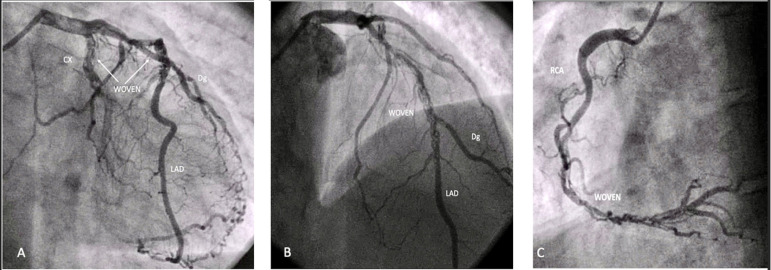
Coronary Angiography - Multivessel woven coronary artery disease. A) LAD affected on its proximal third. CX occluded proximally with collateral filling with the same woven aspect. B) LAD with Swiss cheese aspect on its proximal and middle thirds with the classical "figure of 8". Dg branch affected on its origin. C) RCA affected at the middle and distal thirds; image associated with Swiss cheese aspect. CX=circumflex artery; Dg=diagonal branch of the LAD; LAD=left anterior descending artery; RCA=right coronary artery.

He was then submitted to coronary angiography that showed total occlusion of the circumflex artery on the proximal third, with collateral flow to a posterior marginal branch. The intraluminal filling defect had Swiss cheese aspect characteristic of woven disease. Left anterior descending artery (LAD) had proximal stenosis followed by the same intraluminal filling defect on the second third of the coronary. Diagonal branch originated from the woven segment of the LADs. Right coronary artery (RCA) also showed filling defect on the middle and distal thirds of the coronary and small distal diameter (Figure 2).

The heart team decided that coronary artery bypass grafting (CABG) was the best treatment of choice, considering his anatomy and the risk factors. The patient was successfully treated with on-pump CABG, using the sequence left internal mammary artery to diagonal branch and to LAD (or LIMA-Dg-LAD), left radial artery graft to the obtuse marginal branch of the circumflex artery (or radial-OM), and saphenous vein graft to the right posterior descending artery (or SVG-RPDA).

**Fig. 3:**
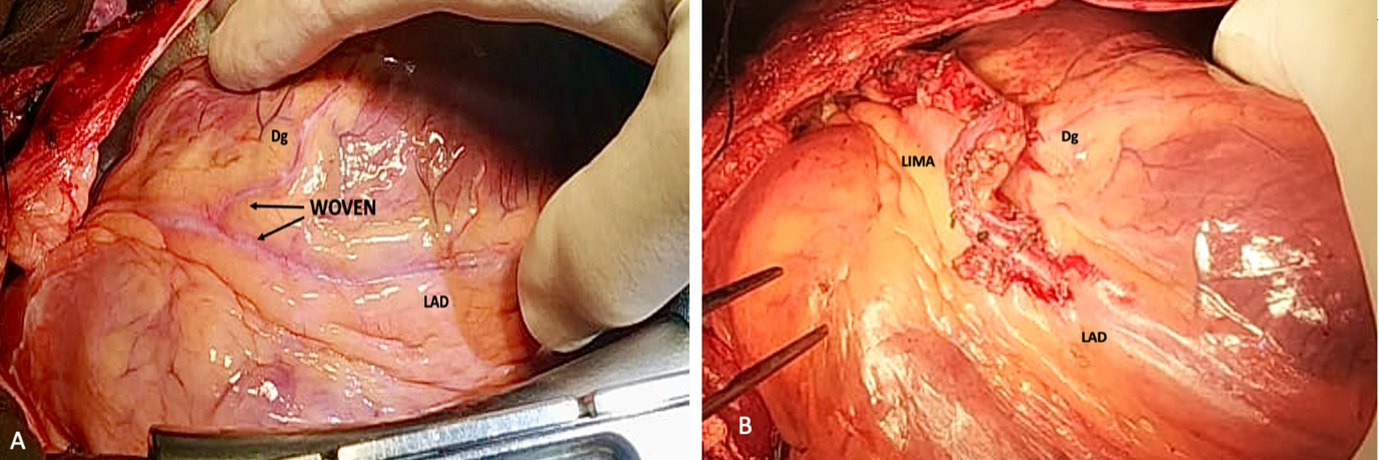
Surgical view of the woven coronary disease. A) LAD and Dg after pericardium opening. B) LIMA sequential to LAD and Dg. Dg=diagonal branch of the LAD; LAD=left anterior descending artery; LIMA=left internal mammary artery.

In a macroscopic view of the coronary, we could notice that the woven segment had a reticular aspect on the coronary edges, but since all anastomoses were performed after the woven diseased segment, we could not see those minor segmentations of the vessel inside the lumen. (Figure 3).

The patient recovered well and was discharged home on postoperative day six, without cardiac or neurological events. His medical treatment consisted of betablockers, aspirin, and statins. After two years of follow-up from surgery, the patient remains asymptomatic.

## QUESTIONS

- What is the woven coronary disease?
- What are the main differential diagnoses of this pathology?
- Should all patients with woven coronary disease be submitted to coronary intervention?
- Does woven coronary disease usually affect one or multiple vessels?

### Discussion of Questions

Question A. Woven coronary disease is a rare conformational coronary disorder. It is characterized by the subdivision of the coronary artery lumen into multiple small independent channels, that merge again into the distal lumen. It usually initiates in the proximal third of the coronary and extends a few centimeters, but there are cases that evolve to almost the full extension of the vessel. It was first described by Sane and Vidaillet, in 1988, as a congenital anomaly, when they observed that the patient's RCA was divided into two major branches which crossed each other in the atrioventricular groove and then fused again near the acute marginal branch as a figure of eight^1^. The etiology and pathogenesis of this disease are still unknown. Initially, some authors believed it was caused by spontaneous coronary dissection or even chronic thrombosis with late recanalization^2^. However, latter publications that studied the coronaries with optical coherence tomography (or OCT) proved that the dissection theory was wrong, since the vessels have multi-fenestrations with an intact wall, without any branches coming out of them^3^. Moreover, histopathological findings of an anomalous segment of an RCA dismissed the dissection hypothesis. It was demonstrated that the lumen was divided in small separated vessels that conserved integrity of their walls, and they did not communicate with each other, showing no signs of dissection, nor thrombus^4^.

Question B. Because of its rarity, it is not always easy to differentiate this condition. Diagnosis must be made for coronary dissection, recanalized thrombus formation, complicated plaque with thrombus formation, and bridging collaterals^2^. As a result of misdiagnosing woven coronary arteries, unnecessary cardiac intervention such as coronary angioplasty may be performed^2^.This ultimately places the patient at an increased risk for arterial damage and other complications^5,6^. In multivessel symptomatic patients, CABG maybe the best treatment option bypassing the diseased segment.

Question C. Since most cases are usually findings on angiograms, optimized medical therapy has been the treatment of choice. Prophylactic use of acetylsalicylic acid in patients with woven coronary anomaly is reasonable. Follow-up of those patients are crucial to understand the need for intervention. There are few studies that reported regular follow-up of the medical therapy patients, but all of them report good outcomes without the need for interventions. The multiple lumen tortuosity may increase the difficulty of percutaneous coronary intervention (PCI), thus CABG may be the best alternate choice if optimized medical therapy fails^7^. There are only two reports of PCI treatment and only three reports of CABG treatment so far, but none of them have woven multiarterial coronary disease. This is the first report to describe a case of a patient with multivessel woven disease, that was successfully treated with CABG.

Question D. There are only 25 cases reported so far and most of them report single vessel disease in young male patients, more frequently affecting the RCA. There are only three cases reported involving two vessels and only one reporting three vessel involvement other than ours.

### Comments

Woven coronary disease is considered a benign condition, since most of the cases reported so far are findings on cardiac angiograms^8^. However, some authors have published reports of patients with angina and acute coronary syndromes related to the affected arteries and even sudden cardiac death^4^.

Although the distal coronary flow is usually preserved, the distance of the thin channels and the twirling of the channels can predispose the vessel to thrombus formation^9^. There are also theories that correlate higher atherosclerosis process at the affected segment due to reduced coronary laminar ﬂow within the thin channels and a greater shear stress. This may ultimately increase the chance of thrombus formation^10^.

This theory is sustained by the previous histological findings showing that those vessels had diffuse mild intimal fibrosis with one of them displaying 80% of luminal stenosis due to a concentric intimal fibrous plaque.

There are also reports that show evidence of silent ischemia with echocardiograms presenting segmented akinesia and scintigraphy with non-viable myocardium related to the woven diseased vessel^10^.

## LEARNING POINTS

- Woven coronary disease is a rare pathology with unknown etiology.
- Although initially considered benign, recent publications report myocardial ischemia caused by the affected vessel.
- In cases with symptomatic multivessel woven coronary disease and when LAD is evolved, surgery maybe the treatment of choice.
- Heart team evaluation should always be recommended.
- Since most patients are asymptomatic, long-term follow-up to understand its behavior is mandatory.
- The use of antiplatelet therapy may be considered to reduce the risk of thrombus formation on the twirling thin channels.

**Table:** 

Authors' roles & responsibilities	
LRPD	Substantial contributions to the conception or design of the work; final approval of the version to be published
LAOD	Final approval of the version to be published
MM	Final approval of the version to be published
ABCM	Substantial contributions to the conception or design of the work; final approval of the version to be published
LAPD	Final approval of the version to be published
FBJ	Final approval of the version to be published
